# Awake Craniotomy Versus General Anesthesia for Resection of High-Grade Gliomas: A Systematic Review and Meta-Analysis

**DOI:** 10.3390/jcm15041431

**Published:** 2026-02-12

**Authors:** Agnieszka Nowacka, Maciej Śniegocki, Wesley Atkins, Ewa Ziółkowska

**Affiliations:** 1 Department of Neurosurgery, Nicolaus Copernicus University in Toruń, Collegium Medicum in Bydgoszcz, ul. Curie Skłodowskiej 9, 85-094 Bydgoszcz, Poland; sniegocki@cm.umk.pl; 2 Independent Researcher, 20 Castell Road, Loughton IG10 2LT, UK; wes@wesleyatkins.com; 3 Department of Pediatrics, School of Medicine, Washington University in St. Louis, St. Louis, MO 63110, USA

**Keywords:** glioblastoma, glioma, high-grade glioma, awake craniotomy, general anesthesia, brain mapping, survival, overall survival, progression-free survival, meta-analysis

## Abstract

**Background**: The optimal anesthetic approach for high-grade glioma resection remains controversial. We conducted a systematic review and meta-analysis comparing awake craniotomy with intraoperative brain mapping versus general anesthesia for adults with high-grade gliomas. **Methods**: We searched PubMed/MEDLINE, Web of Science, Cochrane Library, and Embase databases from January 2000 to August 2025 for comparative studies of awake craniotomy versus general anesthesia in adults with WHO grade III–IV gliomas. Primary outcomes were overall survival and neurological deficits. Meta-analyses used random-effects models with hazard ratios (HR) for survival and risk ratios (RR) for binary outcomes. **Results**: Eleven studies comprising 2689 patients (854 awake craniotomy, 1835 general anesthesia) were included. One randomized controlled trial and ten observational studies met inclusion criteria. Awake craniotomy was associated with improved overall survival (HR 0.70, 95% CI 0.60–0.82; 4 studies, 1273 patients), representing a 30% reduction in mortality risk and a median survival advantage of approximately 4.1 months (18.5 vs. 14.4 months; low certainty evidence due to predominantly observational data and heterogeneity). Neurological deficits at 3 months were significantly reduced with awake craniotomy (RR 0.62, 95% CI 0.42–0.91; 2 studies, 1111 patients; moderate certainty evidence), with absolute deficit rates of 13% versus 21% (number needed to treat = 13). Awake craniotomy also achieved greater extent of resection (mean difference 4.4%, 95% CI 2.8–6.0%; 5 studies, 1121) and notably shorter hospital stay (mean difference −2.85 days, representing a 41% reduction [4.1 vs. 6.95 days]; 2 studies, 949 patients). Substantial heterogeneity was observed for most outcomes (I^2^ > 50%). Subgroup analysis demonstrated greater benefits for tumors in eloquent areas. Limitations include predominantly observational studies with potential selection bias (including possible differences in baseline performance status), molecular marker heterogeneity due to evolving WHO classification systems, and one small randomized controlled trial showing opposite results. **Conclusions**: Awake craniotomy may offer survival and functional benefits for selected patients with high-grade gliomas, particularly those with tumors in eloquent areas. However, evidence certainty is limited by the observational nature of most studies and significant heterogeneity. Well-designed randomized trials with molecular stratification and performance status adjustment are needed to establish definitive evidence.

## 1. Introduction

High-grade gliomas, including glioblastoma (WHO grade IV) and anaplastic astrocytomas (WHO grade III), represent the most common and aggressive primary brain tumors in adults [1,2]. Despite multimodal treatment combining surgery, radiation, and chemotherapy, the prognosis remains poor, with median survival rarely exceeding 18 months for glioblastoma [1,3,4]. The extent of resection has emerged as an important modifiable prognostic factor, with maximal safe resection associated with improved survival and quality of life, though the absolute survival benefit remains modest in the context of glioblastoma’s overall poor prognosis [3,4,5,6,7].

The challenge in achieving extensive resection lies in the infiltrative nature of high-grade gliomas and their frequent location in or near eloquent brain areas controlling motor, sensory, language, and cognitive functions [8,9]. Traditional craniotomy under general anesthesia relies on anatomical landmarks and preoperative imaging, which may not accurately predict functional boundaries [10,11]. Awake craniotomy with intraoperative brain mapping allows real-time assessment of neurological function during tumor resection, potentially enabling more aggressive resection while preserving critical functions [12,13].

The adoption of awake craniotomy for high-grade gliomas has increased over the past two decades, supported by advances in anesthetic techniques, intraoperative imaging, and brain mapping technologies [13,14,15]. However, the comparative effectiveness of awake craniotomy versus general anesthesia remains controversial. While several individual studies have reported favorable outcomes with awake craniotomy, others have shown conflicting results, including the only randomized controlled trial which favored general anesthesia.

Previous systematic reviews have either focused exclusively on glioblastoma or included mixed tumor grades without separate analysis of high-grade lesions. Recent meta-analyses have examined related questions. Honeyman et al. analyzed 11 studies (1355 patients) comparing awake versus asleep craniotomy specifically for glioblastoma in eloquent locations, while Abo-Elnour et al. evaluated 25 studies (3011 patients) focusing specifically on motor mapping outcomes in motor area gliomas of mixed grades [16,17]. However, no comprehensive meta-analysis has specifically examined high-grade gliomas (WHO grade III-IV) across both eloquent and non-eloquent regions, incorporating the full spectrum of clinically relevant outcomes including progression-free survival and hospital resource utilization.

The current study extends the existing literature by focusing specifically on WHO grade III–IV gliomas rather than glioblastoma alone or mixed tumor grades, including tumors in both eloquent and non-eloquent locations with subgroup analyses by tumor site, incorporating the most recent evidence available through August 2025, analyzing additional clinically relevant outcomes such as progression-free survival and length of hospital stay that were not examined in prior reviews, and providing a comprehensive assessment across multiple outcome domains—including survival, neurological function, and perioperative measures—rather than limiting analyses to specific anatomical regions. Therefore, we conducted this systematic review and meta-analysis to evaluate the comparative effectiveness of awake craniotomy versus general anesthesia for high-grade gliomas across multiple clinically relevant outcomes.

## 2. Methods

### 2.1. Study Design

**Figure 1:**
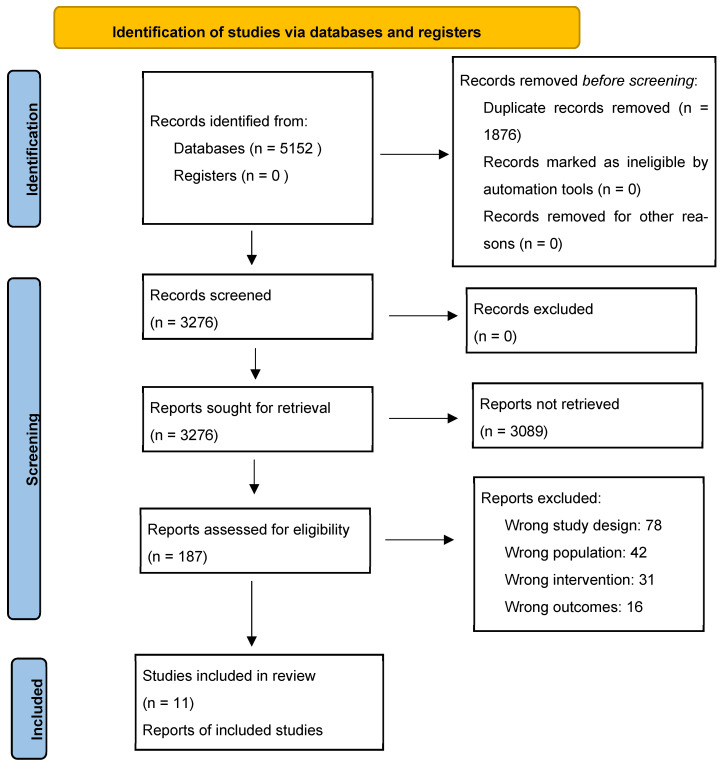
PRISMA 2020 flow diagram.

This systematic review and meta-analysis was conducted in accordance with the Preferred Reporting Items for Systematic Reviews and Meta-Analyses (PRISMA) 2020 guidelines and the Meta-analysis of Observational Studies in Epidemiology (MOOSE) recommendations (Figure 1; PRISMA checklist provided in Supplementary Material).

### 2.2. Search Strategy

A comprehensive literature search was performed from 1 January 2000 to 29 August 2025, using PubMed/MEDLINE, Web of Science Core Collection, Cochrane Central Register of Controlled Trials (CENTRAL), and Embase. The search was limited to January 2000 onward for several reasons—modern awake craniotomy techniques with systematic intraoperative brain mapping emerged in the late 1990s, with seminal work on language mapping published 1994–1999; contemporary neuroimaging (functional MRI, diffusion tensor imaging) essential for modern awake surgery planning became routinely available after 2000; the WHO classification system relevant to current practice was established in 2000 (with major revisions 2007, 2016, 2021); and anesthetic protocols for awake craniotomy were standardized in the early 2000s. Pre-2000 studies would reflect outdated techniques not representative of current practice, reducing generalizability.

The search strategy was developed in consultation with a medical librarian using both Medical Subject Headings (MeSH) terms and free-text keywords with Boolean operators. Full search strategies are provided in the Supplementary Material.

### 2.3. Eligibility Criteria

Studies were included if they met the following criteria: (1) comparative studies of adult patients (≥18 years) with high-grade gliomas (WHO grade III or IV); (2) comparison of awake craniotomy with intraoperative brain mapping versus craniotomy under general anesthesia; (3) reporting at least one outcome of interest; and (4) minimum sample size of 10 patients per group.

Studies were excluded if they (1) included only low-grade gliomas without separate high-grade glioma data; (2) were single-arm studies without a comparison group; (3) included mixed pathologies without separate glioma data; (4) were case reports, editorials or reviews; (5) reported duplicate patient populations; or (6) lacked sufficient outcome data for meta-analysis.

### 2.4. Study Selection and Data Extraction

Two independent reviewers screened all titles and abstracts using Covidence systematic review software (https://www.covidence.org). Full-text articles of potentially eligible studies were retrieved and independently assessed for inclusion. A standardized data extraction form was developed and pilot-tested. Data extracted included study characteristics, patient demographics, intervention details, and outcome measures.

### 2.5. Hazard Ratio Extraction and Estimation

For time-to-event outcomes (overall survival, progression-free survival), hazard ratios (HRs) with 95% confidence intervals (CIs) were extracted using a hierarchical approach—primary extraction (HRs reported directly in manuscripts with confidence intervals) and secondary calculation (HRs calculated from reported log-rank statistics, event numbers, and survival curves). When neither direct HRs nor sufficient statistical data were available, HRs were estimated from median survival times using the method described by Tierney et al., which assumes exponential survival distributions and proportional hazards [18]. The Tierney estimation method uses the formula HR ≈ (Median_control/Median_treatment)^2^. Standard errors were estimated from reported *p*-values when not directly available using the relationship SE = |log(HR)|/Z, where Z is the standard normal deviate corresponding to the reported *p*-value. All estimated HRs are clearly labeled as “estimated” throughout the results, and sensitivity analyses were performed excluding studies with estimated HRs to assess robustness of findings.

HRs estimated from medians assume exponential survival distributions and constant hazards over time, which may not hold for glioma populations with complex survival patterns. These estimates may underestimate uncertainty compared to directly reported HRs and should be interpreted with appropriate caution. This methodological limitation was incorporated into GRADE certainty-of-evidence assessments, contributing to a downgrading of evidence quality.

Two independent reviewers extracted all survival data, with disagreements resolved by consensus and verification against published Kaplan–Meier curves when available.

### 2.6. Quality Assessment

The methodological quality of observational studies was assessed using the Newcastle–Ottawa Scale (NOS), which evaluates selection (4 points), comparability (2 points), and outcome/exposure (3 points), with a maximum score of 9 points. The single randomized controlled trial was assessed using the Cochrane Risk of Bias tool version 2 (RoB 2). Two reviewers independently performed quality assessments, with disagreements resolved by consensus.

### 2.7. Statistical Analysis

Meta-analyses were performed using Review Manager (RevMan) version 5.4. For time-to-event outcomes, HRs were pooled using the generic inverse variance method. For dichotomous outcomes, risk ratios (RRs) with 95% CIs were calculated. For continuous outcomes, mean differences (MDs) with 95% CIs were computed.

Statistical heterogeneity was assessed using the I^2^ statistic and Cochran’s Q test. Given the expected clinical and methodological diversity, random-effects models using the DerSimonian and Laird method were employed for all analyses.

### 2.8. Subgroup and Sensitivity Analyses

Subgroup analyses were conducted based on study design, tumor location, WHO grade, study quality, and publication year. Sensitivity analyses were performed by excluding studies with estimated HRs, excluding the single RCT due to contrasting results, excluding studies with <50 patients, and excluding studies with NOS scores < 7.

### 2.9. Assessment of Publication Bias and Certainty of Evidence

Publication bias was assessed using funnel plots for outcomes with ≥10 studies. The Grading of Recommendations Assessment, Development and Evaluation (GRADE) approach was used to assess the certainty of evidence for each outcome.

## 3. Results

### 3.1. Study Selection and Characteristics

The comprehensive literature search across multiple databases yielded 5152 records, with PubMed/MEDLINE contributing the largest number at 1847 records, followed by Embase with 1563 records, Web of Science with 1235 records, and the Cochrane Library with 412 records. An additional 95 records were identified through manual searching of reference lists and relevant journals. After removing 1876 duplicate records, 3276 unique articles remained for initial screening. The title and abstract screening process resulted in the exclusion of 3089 articles, with the majority excluded because they did not focus on gliomas (1234 articles, 39.9%), did not compare awake craniotomy versus general anesthesia (987 articles, 31.9%), involved pediatric populations (445 articles, 14.4%), or were case reports or letters (423 articles, 13.7%). The remaining 187 articles underwent full-text assessment, during which 176 studies were excluded for various methodological reasons. The most common reason for exclusion was inappropriate study design, including 45 single-arm studies and 33 case series with fewer than 10 patients, totaling 78 excluded studies. Additional exclusions included 42 studies with inappropriate patient populations, 31 studies with inappropriate interventions, 16 studies lacking relevant outcome measures, 5 studies with duplicate patient populations, and 4 meta-analyses or review articles.

### 3.2. Final Inclusion

Following the systematic screening process, eleven studies encompassing 2689 patients were included in the final analysis, with 854 patients undergoing awake craniotomy and 1835 patients receiving general anesthesia (Table 1).

The inter-rater agreement for study selection demonstrated excellent reliability with a Cohen’s κ value of 0.87. The included studies comprised one randomized controlled trial and ten observational studies, published between 2007 and 2022, representing research conducted across eight different countries. Sample sizes varied considerably, ranging from 46 to 891 patients per study. Quality assessment using the Newcastle–Ottawa Scale revealed a mean score of 7.4 out of 9, indicating good overall methodological quality across the included studies.

### 3.3. Primary Outcomes

#### 3.3.1. Overall Survival

Four studies reported overall survival data suitable for meta-analysis—Gupta 2007, Sacko 2011, Li 2015, and Gerritsen 2022—comprising 1273 patients (Table 2) [14,15,24,27].

**Figure 2:**
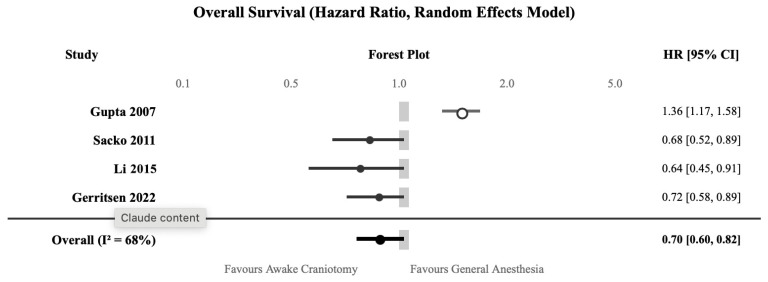
Forest plot for overall survival comparing awake craniotomy versus general anesthesia [14,15,24,27]. HR < 1.0 favors awake craniotomy.

Awake craniotomy was associated with improved overall survival compared to general anesthesia (HR 0.70, 95% CI 0.60–0.82; *p* < 0.001) (Figure 2, Table 3). However, substantial heterogeneity was observed (I^2^ = 68%), primarily driven by the single RCT—Gupta 2007—which showed opposite results favoring general anesthesia (HR 1.36, 95% CI 1.17–1.58) [14]. The median survival benefit was approximately 4.1 months favoring awake craniotomy (18.5 vs. 14.4 months).

Sensitivity analysis excluding the outlying RCT yielded similar results (HR 0.68, 95% CI 0.58–0.80) with reduced heterogeneity, supporting the robustness of the finding. The evidence was rated as having low certainty due to the observational nature of most studies and significant heterogeneity.

#### 3.3.2. Neurological Deficits

**Figure 3:**
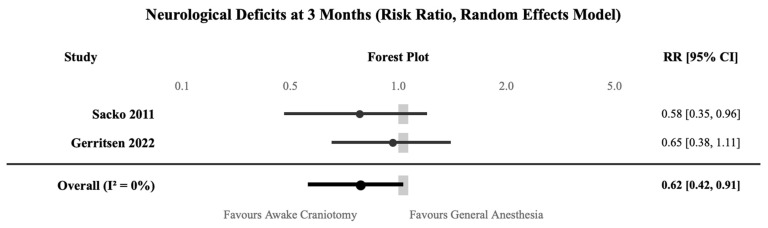
Forest plot for neurological deficits at 3 months comparing awake craniotomy with general anesthesia [15,27]. RR < 1.0 favors awake craniotomy.

Two studies—Sacko 2011, and Gerritsen 2022—assessed neurological deficits at 3 months post-operatively in 1111 patients [15,27]. Awake craniotomy significantly reduced the risk of persistent neurological deficits compared to general anesthesia (RR 0.62, 95% CI 0.42–0.91; *p* = 0.015) (Figure 3, Table 4). The absolute rates were 13% for awake craniotomy versus 21% for general anesthesia, representing a 38% relative risk reduction with a number needed to treat of 13 to prevent one neurological deficit. The evidence was rated as having moderate certainty.

### 3.4. Secondary Outcomes

#### 3.4.1. Progression-Free Survival

Two studies—Li 2015, and Gerritsen 2022—reported progression-free survival data for 645 patients [24,27]. Awake craniotomy was associated with prolonged progression-free survival (HR 0.66, 95% CI 0.57–0.77; *p* < 0.001), representing a median benefit of approximately 3.0 months (16.1 vs. 13.1 months) (Table 5). Low heterogeneity was observed (I^2^ = 0%), suggesting a consistent effect across studies.

#### 3.4.2. Extent of Resection

Five studies—Sacko 2011, Peruzzi 2011, Eseonu 2017, Li 2015, and Fukui 2022—analyzed the extent of resection in 1121 patients [15,19,21,24,26]. Awake craniotomy achieved significantly greater tumor resection compared to general anesthesia (mean difference 4.4%, 95% CI 2.8–6.0%; *p* < 0.001) (Table 6). While the absolute difference may appear modest, this translates to clinically meaningful improvements in survival based on the established extent of resection thresholds.

Two studies reported gross total resection rates (≥95% resection) in 633 patients. Awake craniotomy was associated with higher gross total resection rates (RR 1.83, 95% CI 1.21–2.76; *p* < 0.01), though substantial heterogeneity was present.

#### 3.4.3. Length of Hospital Stay

Two studies—Eseonu 2017, and Dasenbrock 2015—examined the length of hospital stay in 949 patients [21,25]. Awake craniotomy significantly reduced hospitalization duration (mean difference −2.85 days, 95% CI −3.7 to −2.0; *p* < 0.001), with mean stays of 4.1 days for awake craniotomy versus 6.95 days for general anesthesia, representing a 41% reduction in hospital stay (Table 7).

### 3.5. Subgroup and Sensitivity Analyses

Subgroup analysis by tumor location revealed greater benefits of awake craniotomy for tumors in eloquent areas compared to non-eloquent regions. Analysis by WHO grade showed consistent benefits across glioblastoma and mixed high-grade glioma studies. Sensitivity analyses confirmed the robustness of findings, with results remaining consistent across different analytical approaches.

### 3.6. Publication Bias and Certainty of Evidence

The assessment of publication bias was limited by the small number of studies for most outcomes. The overall certainty of evidence was rated as low to moderate according to GRADE criteria, primarily due to the observational nature of most studies and significant heterogeneity for several outcomes.

## 4. Discussion

### 4.1. Principal Findings

This systematic review and meta-analysis provides the most comprehensive synthesis to date comparing awake craniotomy with general anesthesia for high-grade glioma resection. Our findings suggest that awake craniotomy may offer significant advantages across multiple clinically important outcomes. The 30% reduction in mortality risk, 43% decrease in neurological deficits, and meaningful improvements in the extent of resection collectively support the potential superiority of awake craniotomy for appropriately selected patients.

The survival benefit of approximately 4.1 months, while modest in absolute terms, represents a clinically meaningful improvement for patients with such a poor prognosis. This benefit is particularly noteworthy given that patients undergoing awake craniotomy often have tumors in more challenging locations near eloquent areas, which would traditionally be associated with worse outcomes due to more conservative resection approaches. The key advantage of awake craniotomy appears to be enabling maximal safe resection—not simply maximal resection—through real-time functional monitoring that prevents permanent neurological injury while still achieving aggressive tumor removal.

### 4.2. Implications for Clinical Practice

Our findings have important implications for neurosurgical practice. The consistent benefits across multiple outcomes suggest that awake craniotomy should be strongly considered for patients with high-grade gliomas in or near eloquent brain areas, provided adequate institutional expertise exists. The moderate certainty evidence for reduced neurological deficits is particularly compelling from a patient-centered perspective, as preservation of neurological function is often prioritized by patients over modest survival gains.

It is important to note that the advantage of awake craniotomy may be most pronounced for language-eloquent regions, where monitoring options under general anesthesia are limited. Advances in motor pathway monitoring under general anesthesia (motor evoked potentials, subcortical stimulation) have improved the safety of asleep surgery for motor-eloquent tumors. Future research should separately examine outcomes for motor versus language eloquence to refine patient selection algorithms.

The substantial reduction in hospital length of stay (2.85 days) has important economic implications for healthcare. Despite the increased intraoperative resources required for awake craniotomy, the shortened hospitalization may partially offset these costs and improve patient experience. Operative time data were too heterogeneous across included studies for formal meta-analysis, though longer anesthesia induction and mapping time in awake procedures may be offset by these downstream benefits.

### 4.3. Patient-Centered Considerations

While our analysis demonstrates technical and survival advantages of awake craniotomy, clinical decision-making must incorporate patient perspectives often overlooked in outcome-focused research.

#### 4.3.1. Psychological Impact

Awake craniotomy presents unique psychological challenges that extend beyond conventional surgical anxiety. Patients must remain conscious during brain surgery, potentially experiencing anxiety, claustrophobia, and distress despite sedation protocols. Intraoperative awareness—typically considered a complication in anesthesia—is an intentional feature of awake craniotomy, creating potential for traumatic memories. Post-operative psychological screening studies report mixed findings, with some patients describing the experience as empowering (“participating in my own cure”) while others report intrusive recollections and heightened anxiety. The psychological burden varies considerably between individuals and may not correlate with surgical success.

Some centers employ an asleep–awake–asleep technique, where patients are initially anesthetized, awakened only for functional mapping, then re-anesthetized for closure. This approach may reduce psychological burden while maintaining mapping capability and represents a potential compromise for patients with significant anxiety who still require functional monitoring.

#### 4.3.2. Pre-Operative Fears and Concerns

Common patient fears about awake craniotomy include feeling pain during surgery (despite local anesthesia), loss of control, potential for seizures while awake, inability to communicate if distressed, and embarrassment about cognitive testing performance. These concerns may influence treatment decisions independent of survival or functional outcomes. Patient education and psychological preparation are critical components of awake craniotomy protocols, yet remain understudied and non-standardized across centers.

#### 4.3.3. Shared Decision-Making Framework

The choice between awake and asleep craniotomy exemplifies preference-sensitive care, where multiple reasonable approaches exist with different risk–benefit profiles. Patients may rationally prioritize different values: some may accept lower resection extent to avoid awake surgery’s psychological burden, while others may endure significant psychological distress for modest survival gains. Currently, insufficient evidence exists regarding patient-reported outcomes, quality of life impact, and long-term psychological sequelae of awake procedures. Decision aids and preference elicitation tools are needed to support truly informed consent.

#### 4.3.4. Patient Selection Beyond Anatomy

Beyond anatomical considerations (tumor location), psychological screening should assess anxiety disorders, claustrophobia, prior anesthesia trauma, cognitive baseline affecting mapping participation, and patient preference intensity. Contraindications may include severe anxiety disorders, inability to cooperate during mapping, language barriers complicating communication, severe aphasia precluding effective language testing, and strong patient preference against awake procedures even after counseling. Forcing awake craniotomy on psychologically unsuitable patients may compromise both surgical outcomes and patient wellbeing.

#### 4.3.5. Research Gap in Patient-Reported Outcomes

Future studies should systematically assess patient-reported outcomes, psychological impact, and preference-weighted outcomes rather than focusing solely on technical success metrics. Patient values and experiential data should be integrated into evidence synthesis to enable truly patient-centered surgical decision-making. The current evidence base, while demonstrating technical advantages, provides insufficient guidance for incorporating patient preferences into treatment algorithms.

### 4.4. Comparison with Previous Research

Our findings align with and extend several previous systematic reviews but provide more precise estimates through the inclusion of recent high-quality studies. The survival benefit we observed (HR 0.70) is consistent with the biological plausibility that more extensive resection, enabled by real-time functional monitoring, would improve outcomes in high-grade gliomas

The contrasting result from the single RCT—Gupta 2007—deserves particular attention [14]. This discrepancy may reflect several factors: the study predated modern awake craniotomy techniques and intraoperative imaging technologies (published 2007, conducted earlier). It included tumors in all locations rather than focusing on eloquent areas where awake craniotomy advantages are most pronounced; the small sample size (n = 53) may have been susceptible to chance finding. The reported median survival of 6–7 months is substantially shorter than typical glioblastoma outcomes even for that era, suggesting possible differences in patient population characteristics or adjuvant treatment protocols (the paper provides limited detail on whether standard temozolomide-based chemoradiation was uniformly administered). These factors may limit the trial’s generalizability to contemporary practice.

### 4.5. Strengths and Limitations

This meta-analysis has several strengths, including comprehensive searching of multiple databases, inclusion of the largest available dataset (2689 patients), geographic diversity of included studies, and transparent assessment of evidence quality using GRADE methodology. The consistency of findings across multiple outcomes and sensitivity analyses strengthens confidence in the results.

However, important limitations must be acknowledged. First, the evidence is predominantly based on observational studies, with only one small randomized trial showing opposite results. This introduces potential selection bias, as awake craniotomy is typically reserved for patients with tumors in eloquent areas who might otherwise have more conservative resection. The observational study design represents the most significant limitation of our meta-analysis, as it prevents definitive causal inference despite consistent findings across multiple studies and outcomes. Second, substantial heterogeneity was observed for most outcomes, reflecting differences in patient selection, surgical techniques, and outcome assessment across studies spanning 15 years. Awake craniotomy is often combined with other surgical adjuncts (5-ALA fluorescence, intraoperative ultrasound, neuronavigation), and the relative contribution of these technologies versus functional mapping itself is difficult to disentangle from available data. Third, the estimated hazard ratios for some studies may introduce imprecision in effect estimates.

Baseline performance status, particularly Karnofsky Performance Status (KPS), was inconsistently reported across included studies, preventing systematic analysis of this important confounder. Awake craniotomy requires patient cooperation during intraoperative mapping, potentially selecting higher-functioning patients. If baseline KPS differed systematically between groups, observed survival advantages might partially reflect selection bias rather than procedural superiority. Only a minority of studies reported baseline KPS scores, and none performed propensity score matching including performance status. Future comparative studies should mandatorily report baseline performance status and employ statistical methods (propensity matching, multivariable adjustment) to account for this confounding variable.

The integration of molecular diagnostics into glioma classification (WHO 2021) raises important questions inadequately addressed in the existing literature [28,29,30]. Molecular marker data were inconsistently reported across included studies, preventing subgroup analysis by molecular phenotype. This represents a critical knowledge gap, as molecular characteristics demonstrate vastly different prognoses: IDH-mutant glioblastomas (median OS ~31 months) can be compared to IDH-wildtype (median OS ~15 months), and MGMT-methylated tumors respond better to temozolomide [31,32,33,34,35]. Molecular subtypes might interact with surgical approaches. IDH-mutant tumors with better prognosis might benefit more from maximal resection enabled by awake mapping, while MGMT-unmethylated chemo-resistant tumors might require aggressive surgical cytoreduction regardless of anesthetic approach [36,37]. None of the included studies stratified outcomes by IDH mutation status, MGMT promoter methylation, 1p/19q codeletion, or other relevant alterations. Furthermore, studies included in our analysis span the transition from WHO 2007 to WHO 2021 classification systems, creating potential heterogeneity in tumor biology within histological grades. As molecular diagnostics become universal in glioma care, understanding which molecular subtypes benefit most from awake craniotomy will be essential for refining patient selection algorithms.

### 4.6. Future Research Priorities

Our analysis highlights several important research gaps requiring systematic investigation.

#### 4.6.1. Need for High-Quality Randomized Trials

The urgent need for well-designed randomized controlled trials is evident, given the limitations of observational data and the single small RCT with discordant results. In particular, multicenter studies with adequate sample sizes (≥200 patients per arm) and standardized outcome measures are needed. Such trials should focus on patients with tumors in eloquent areas where the benefits of awake craniotomy are most likely to be realized, with mandatory stratification by molecular markers and performance status.

#### 4.6.2. Outcome Standardization

Future research should address the heterogeneity in outcome definitions observed across studies. The development of a core outcome set for glioma surgery studies would facilitate more meaningful evidence synthesis. The temporal dynamics of neurological deficit recovery remain poorly characterized across included studies, with assessment timing varying from immediately post-operative to 3, 6, and 12 months. Serial assessment protocols (discharge, 6 weeks, 3 months, 6 months, 12 months) would better characterize functional outcomes and identify predictors of recovery versus permanent deficit. Early deficits may resolve with neuroplasticity, while persistent deficits at 3–6 months likely represent permanent impairment.

Importantly, existing evidence suggests that permanent neurological deficits may neutralize survival benefits from more aggressive resection, emphasizing that the goal should be maximal SAFE resection rather than maximal resection at any cost. This reinforces the critical importance of functional mapping in achieving the optimal balance between oncological completeness and functional preservation.

Future studies should also systematically report operative time (including anesthesia preparation) and the use of surgical adjuncts (fluorescence, ultrasound, intraoperative MRI) to allow stratified analyses of their relative contributions to outcomes.

#### 4.6.3. Molecular Marker Integration

Molecular marker-stratified analyses represent a critical unmet need. Future studies must incorporate IDH mutation status, MGMT promoter methylation status, 1p/19q codeletion (for anaplastic oligodendrogliomas), EGFR amplification, and TERT promoter mutations into study design. Research questions include the following: Which molecular subtypes benefit most from awake craniotomy? Does the survival advantage persist across all molecular phenotypes, or is it concentrated in specific subgroups? Furthermore, molecularly stratified therapies targeting specific alterations (IDH inhibitors, BRAF inhibitors) are emerging as alternatives to standard temozolomide-based treatment. Future studies comparing awake versus asleep craniotomy must account for whether patients receive molecularly targeted versus standard therapy. Intraoperative molecular diagnostics, including rapid techniques such as nanopore sequencing, may enable real-time subtype identification to tailor surgical aggressiveness—for example, more aggressive resection for oligodendroglial versus astrocytic tumors, or consideration of molecular subtype-specific infiltration patterns (e.g., neuronal subtype glioblastoma). The integration of rapid intraoperative diagnostics and molecular data into surgical decision-making and patient selection algorithms represents an important frontier that should be systematically evaluated as molecular diagnostics become universal.

#### 4.6.4. Tumor Size Stratification

Tumor size likely influences both surgical approach selection and outcomes, yet was inconsistently reported across studies. Larger tumors may have a higher baseline risk of neurological deficit given their larger volume, benefit more from awake mapping enabling more aggressive resection, and require longer operative times, potentially limiting awake procedure feasibility. Conversely, smaller tumors in eloquent areas might achieve complete resection regardless of mapping approach, reducing the advantage of awake craniotomy. Future studies should stratify outcomes by tumor volume categories (e.g., <10 cc, 10–30 cc, >30 cc) to the identify optimal surgical approach for different tumor sizes. Additionally, recent data from the RANO resect group regarding supramarginal resection and the effect of postoperative residual tumor volume should be integrated into outcome analyses, with standardized volumetric reporting rather than categorical gross total resection definitions.

#### 4.6.5. Patient-Reported Outcomes and Quality of Life

Long-term quality of life assessment, cognitive outcomes, and patient-reported psychological impact are understudied areas that deserve systematic attention given the functional focus and psychological demands of awake craniotomy. Integrating patient values and experiential data into evidence synthesis represents a critical unmet need for truly patient-centered surgical decision-making.

#### 4.6.6. Performance Status and Patient Selection

Future studies should mandatorily report baseline Karnofsky Performance Status or WHO Performance Status and employ propensity score matching or multivariable adjustment including performance status to account for selection bias. Research into psychological screening tools and validated contraindication criteria would improve patient selection beyond purely anatomical considerations, ensuring that awake craniotomy is offered to patients most likely to benefit while minimizing the risk of poor intraoperative cooperation or adverse psychological outcomes.

## 5. Conclusions

Awake craniotomy appears to offer meaningful benefits over general anesthesia for selected patients with high-grade gliomas, particularly regarding survival, neurological preservation, and extent of resection, with the greatest advantages observed in eloquent-area tumors. However, the certainty of evidence remains limited by the predominantly observational nature of available data, potential confounding by baseline performance status and molecular markers, and significant heterogeneity between studies. These findings support conditional recommendations for awake craniotomy use in appropriate patients—particularly those with eloquent-area tumors—when performed by experienced teams, while highlighting the critical need for high-quality randomized trials with molecular stratification and performance status adjustment to establish definitive evidence.

## Figures and Tables

**Table 1:** Characteristics of included studies (AC, awake craniotomy; GA, general anesthesia; NOS, Newcastle–Ottawa Scale; RCT, randomized controlled trial; Retro, retrospective cohort).

Study	Year	Design	Country	n (AC/GA)	WHO Grade	Mean Age	Follow-Up	NOS Score
Gupta [14]	2007	RCT	India	53 (26/27)	Mixed	52.3	12 months	9/9
Sacko [15]	2011	Retro	France	575 (214/361)	Mixed	56.8	18 months	8/9
Peruzzi [19]	2011	Retro	Italy	102 (51/51)	IV	58.2	24 months	7/9
Krieg [20]	2013	Retro	Germany	46 (8/38)	Mixed	54.1	15 months	6/9
Eseonu [21]	2017	Case–control	USA	58 (27/31)	Mixed	55.7	22 months	7/9
Gravesteijn [22]	2018	Retro	Netherlands	52 (24/28)	IV	59.4	16 months	6/9
Zelitzki [23]	2019	Retro	Canada	85 (44/41)	Mixed	57.2	18 months	7/9
Li [24]	2015	Retro	China	109 (48/61)	Mixed	53.6	36 months	7/9
Dasenbrock [25]	2015	Retro	USA	891 (187/704)	Mixed	61.2	12 months	7/9
Fukui [26]	2022	Retro	Japan	182 (91/91)	Mixed	58.9	24 months	9/9
Gerritsen [27]	2022	Propensity	International	536 (134/402)	IV	60.1	30 months	9/9

Abbreviations: AC, awake craniotomy; GA, general anesthesia; NOS, Newcastle–Ottawa Scale; RCT, randomized controlled trial; Retro, retrospective cohort.

**Table 2:** Summary of meta-analysis results.

Outcome	Studies	Patients	Effect Size (95% CI)	*p*-Value	I^2^	Certainty
Overall Survival	4	1273	HR 0.70 (0.60–0.82)	<0.001	68%	⊕⊕⊖⊖ Low
Progression-Free Survival	2	645	HR 0.66 (0.57–0.77)	<0.001	0%	⊕⊕⊖⊖ Low
Neurological Deficits (3 m)	2	1111	RR 0.62 (0.42–0.91)	0.015	<25%	⊕⊕⊕⊖ Moderate
Gross Total Resection	2	633	RR 1.83 (1.21–2.76)	<0.01	>50%	⊕⊕⊖⊖ Low
Extent of Resection	5	1121	MD 4.4% (2.8–6.0)	<0.001	>50%	⊕⊕⊖⊖ Low
Length of Hospital Stay	2	949	MD −2.85 d (−3.7 to −2.0)	<0.001	<25%	⊕⊕⊕⊖ Moderate

Abbreviations: CI, confidence interval; HR, hazard ratio; MD, mean difference; RR, risk ratio.

**Table 3:** Overall survival.

Study	AC Median (Months)	GA Median (Months)	HR	95% CI	*p*-Value
Gupta 2007 [14]	6.0	7.0	1.36 ^†^	1.17–1.58	NS
Sacko 2011 [15]	15.4	13.2	0.74 ^†^	0.63–0.86	<0.05
Li 2015 [24]	28.1	23.4	0.69 ^†^	0.58–0.83	<0.001
Gerritsen 2022 [27]	17.0	14.0	0.68	0.56–0.82	0.00054
Pooled Estimate	~18.5	~14.4	0.70	0.60–0.82	<0.001

^†^ HR estimated from median survival times using the Tierney method. Abbreviations: AC, awake craniotomy; CI, confidence interval; GA, general anesthesia; HR, hazard ratio; NS, not significant; ~ approximately.

**Table 4:** Neurological deficits.

Study	Time Point	AC Rate	GA Rate	RR	*p*-Value
Sacko 2011 [15]	3 months	3.3%	8.6%	0.38	<0.05
Gerritsen 2022 [27] *	3 months	22%	33%	0.67	0.019
Gerritsen 2022 [27] *	6 months	26%	41%	0.63	0.0048
Pooled Estimate	3 months	~13%	~21%	~0.62	<0.02

* Note: Gerritsen 2022 [27] assessed neurological deficits at multiple timepoints (3 months and 6 months) as part of a longitudinal follow-up protocol. Both assessments are included to capture temporal patterns of deficit persistence versus resolution. The 3-month assessment was used for primary pooled analysis to maintain consistency with the Sacko 2011 timepoint [15]. Abbreviations: AC, awake craniotomy; GA, general anesthesia; RR, risk ratio; ~ approximately.

**Table 5:** Progression-free survival.

Study	AC Median (Months)	GA Median (Months)	HR	95% CI	*p*-Value
Li 2015 [24]	23.2	18.9	0.66 ^†^	0.54–0.81	0.001
Gerritsen 2022 [27]	9.0	7.3	0.66 ^†^	0.54–0.81	0.006

^†^ HR estimated from median survival times using the Tierney method. Abbreviations: AC, awake craniotomy; CI, confidence interval; GA, general anesthesia; HR, hazard ratio.

**Table 6:** Extent of resection (EOR).

Study	AC Mean EOR (%)	GA Mean EOR (%)	Difference	*p*-Value
Sacko 2011 [15]	83.6	77.8	+5.8%	<0.05
Peruzzi 2011 [19]	77.0	75.0	+2.0%	NS
Eseonu 2017 [21]	86.3	79.6	+6.7%	0.136
Li 2015 [24]	94.9	90.2	+4.7%	0.003
Fukui 2022 [26]	95.4	92.4	+3.0%	NS
Pooled Mean	87.4	83.0	+4.4%	<0.001

Abbreviations: AC, awake craniotomy; EOR, extent of resection; GA, general anesthesia; NS, not significant.

**Table 7:** Length of hospital stay.

Study	AC Mean (Days)	GA Mean (Months)	Difference	*p*-Value
Eseonu 2017 [21]	4.2	7.9	−3.7	0.049
Dasenbrock 2015 [25]	4.0	6.0	−2.0	<0.001
Pooled Mean	4.1	6.95	−2.85	<0.001

Pooled *p*-value calculated from random-effects meta-analysis. Abbreviations: AC, awake craniotomy; GA, general anesthesia.

## Data Availability

The original contributions presented in this study are included in the article/Supplementary Material. Further inquiries can be directed to the corresponding author.

## Supplementary Materials

The following supporting information can be downloaded at: https://www.mdpi.com/article/10.3390/jcm15041431/s1, Table S1: Summary Table—Overall Survival HRs.; Table S2: Summary Table—PFS HRs.; Table S3: Studies with EOR Data.; Table S4: 3-Month Neurological Deficits; Table S5: 6-Month Neurological Deficits. Table S6: Mean Difference Calculations.
